# Standing up heals: A qualitative analysis of participants' daily reflections from Emotion-Focused Training for Helping Professions (EFT-HP)

**DOI:** 10.1371/journal.pone.0354851

**Published:** 2026-10-29

**Authors:** Silvia Aziriová, Júlia Halamová, Timotea Vráblová, Katarína Greškovičová

**Affiliations:** Institute of Applied Psychology, Faculty of Social and Economic Sciences, Comenius University in Bratislava, Bratislava, Slovakia; Universiti Sains Malaysia - Kampus Kesihatan, MALAYSIA

## Abstract

**Background:**

Helping professionals face elevated risks of compassion fatigue and burnout, yet the daily processes through which emotional transformation occurs during therapeutic interventions remain poorly understood. This study examined how participants’ daily reflections shifted across the Emotion-Focused Training for Helping Professions (EFT-HP), analyzed through three complementary theoretical frameworks: Emotion-Focused Therapy's inner dialogue model (self-criticism, self-compassion, self-protection), Compassion-Focused Therapy's three-systems framework (threat, drive, soothing), and Ekman's basic emotion theory (primary emotions).

**Method:**

Two hundred twenty-five helping professionals (88.0% female; M age = 38.7 years) from healthcare, social work, psychology, and education began the 14-day online EFT-HP intervention. Primary analyses focused on the 146 participants who completed at least 9 of the 14 days (completers), whose daily written reflections yielded 7,307 coded reflection units. Reflections were analyzed with directed content analysis and frequency quantification, applying non-exclusive, AI-assisted coding within three theoretical frameworks—EFT inner-dialogue categories, CFT emotion-regulation systems, and Ekman's discrete emotions—with a verbatim textual evidence excerpt retained for every assigned code.

**Results:**

Across the intervention, participants’ reflections shifted from threat- and self-criticism-related content toward soothing and self-compassion. Within the EFT inner-dialogue framework, Self-Compassion was the most frequent category (17.9% of units) and was sustained throughout (Day-13 peak 30.8%), while Self-Criticism fell from 14.3% on Day 1 to 3.9% on Day 14 (73% relative decrease) and Self-Protection peaked on Day 6 (11.3%). Within the CFT emotion-regulation framework, the soothing system was the most frequently coded system overall (31.8% of units), followed by drive (20.4%); over the 14 days the threat system declined from 35.4% on Day 1 to 7.0% on Day 14 (80% relative decrease). Within Ekman's discrete-emotion framework, specific emotions were named less frequently: Fear declined from 2.7% to 0.8%, Anger peaked on Day 6 (15.0%), Joy rose to a Day-13 peak (14.8%), and Love was comparatively prominent (11.9% overall, Day-5 peak).

**Conclusions:**

Across the 14-day intervention, participants’ daily reflections showed substantial shifts in emotional content, characterized by marked self-criticism and threat reduction alongside sustained self-compassion and soothing activation. Triangulated findings across three analytical frameworks: EFT inner dialogue, CFT emotion systems, and Ekman's primary emotions, are consistent with the emotional-transformation processes proposed by EFT-HP and with findings from previous controlled trials. Because the three frameworks were applied to the same reflection data, their convergence reflects analytic (theoretical) triangulation rather than independent confirmation.

## Introduction

Helping professionals routinely engage with others’ suffering as a core component of their work, yet the psychological mechanisms that enable some practitioners to sustain this engagement while others experience occupational distress remain insufficiently understood [1]. The prevalence of compassion fatigue ranges from 7% to 40% among emergency care and intensive care workers, from 22% to 45% among nurses generally, and reaches 60–76% among oncology healthcare professionals [2–4]. Distinct from burnout, compassion fatigue—a term introduced by Joinson [5] to describe the exhaustion specific to caregiving work—can emerge acutely following intense empathic engagement with distressed clients. Figley [1] conceptualized compassion fatigue as an inherent occupational hazard of emotionally demanding work. Stamm's [6] Professional Quality of Life model provides a more comprehensive framework, positing that helping professionals simultaneously experience positive outcomes (compassion satisfaction) alongside negative consequences (compassion fatigue). The balance between these dimensions has significant implications: elevated compassion fatigue is associated with diminished quality of care and increased workforce attrition [7,8].

Understanding the mechanisms underlying compassion fatigue and compassion satisfaction requires theoretically grounded intervention approaches. The present study examines an Emotion-Focused Therapy intervention adapted for helping professionals (EFT-HP), drawing on complementary frameworks that illuminate how emotional transformation may protect against compassion fatigue.

### The theoretical framework

Emotion-Focused Therapy [9] provides the primary theoretical foundation for understanding how emotional transformation occurs. EFT is grounded in the view that emotion is foundational in the construction of the self, with personal meaning emerging through the self-organization and explication of one's emotional experience [10].

Pascual-Leone and Greenberg's [11] sequential model of emotional processing is particularly relevant. According to this model, emotional transformation occurs through specific sequences: individuals move from undifferentiated global distress through secondary reactive emotions, access primary maladaptive emotions (“core pain”), and finally by connecting with unmet needs, locate primary adaptive emotions such as assertive anger or self-protection, and self-compassion [12]. Research across 24 studies and over 440 clinical cases demonstrates that this emotional transformation process shows large effects in predicting positive treatment outcomes [12].

Crucially, the EFT model identifies self-compassion and self-protection (assertive anger) as particularly important for transforming self-criticism [13], which is considered a key predictor of compassion fatigue across various helping professions [14]. Effective intervention must include not only soothing self-compassion but also assertive self-protective responses that enable individuals to stand up for themselves against their inner critic [9,12]. This dual emphasis on compassion and protection provides the theoretical foundation for the EFT-HP intervention examined in this study.

Gilbert’s [15,16] Compassion-Focused Therapy (CFT) offers a complementary neurobiological framework, proposing three interacting emotion regulation systems: the threat system (detecting danger, generating anxiety and shame), the drive system (motivating achievement and goal pursuit), and the soothing system (generating feelings of safeness and social connection). Activation of the soothing system can downregulate threat system activity, providing a neurobiological pathway through which self-compassion produces its benefits [16,17]. This framework enriches the primary EFT model by explaining how interventions produce effects through strengthening access to the soothing system.

Beyond these therapeutic frameworks, Ekman's [18,19] basic emotion theory provides a complementary lens for understanding emotional transformation at the most granular level. This theory posits that humans possess a set of universal, biologically-based primary emotions, fear, anger, disgust, contempt, sadness, surprise, and joy, that evolved to serve adaptive functions and are recognizable across cultures. These discrete emotions represent fundamental building blocks of emotional experience, each associated with distinct physiological signatures, facial expressions, and action tendencies [20]. While EFT and CFT frameworks capture broader patterns of self-relating and system activation, discrete emotion analysis can reveal which specific emotions decrease (e.g., fear, anger) and which increase (e.g., joy) as participants progress through intervention. For the present study, we extended this framework to include love as a primary positive emotion given its relevance to compassion and self-compassion [21,22].

### Interventions for compassion in helping professionals

A growing body of research has examined interventions designed to reduce compassion fatigue and enhance compassion satisfaction. A recent systematic review and meta-analysis of 82 intervention studies and 11 randomized controlled trials found that psychological interventions are highly effective in reducing compassion fatigue among helping professionals (SMD = −0.95; 95% CI, −1.63 to −0.27; *p* = .006), with online-delivered interventions showing particularly strong effects (SMD = −1.22 [2]). Earlier reviews similarly documented significant reductions in compassion fatigue following psychological interventions [23]. Several interventions have demonstrated effects on both reducing compassion fatigue and increasing compassion satisfaction [24,25].

The Emotion-Focused Training for Helping Professionals (EFT-HP [26,27]) represents a theoretically grounded approach uniquely integrating EFT principles of emotional transformation with compassion fatigue prevention and compassion satisfaction facilitation. Two RCTs have demonstrated its quantitative effectiveness: Halamová et al. [26] found significant reductions in secondary traumatic stress, burnout, and self-criticism, with increased self-compassion maintained at 2-month follow-up; Halamová et al. [27] replicated these findings, demonstrating improved compassion satisfaction alongside reduced compassion fatigue.

### The research aim

Although quantitative evidence supports the effectiveness of EFT-HP, qualitative research examining participants’ lived experiences during the intervention process remains absent. The predominant methodological approach in compassion fatigue intervention research relies on pre-post quantitative designs, which establish intervention efficacy but provide limited insight into the mechanisms and temporal processes through which change occurs [28]. Daily-level analysis would provide unprecedented insight into the temporal dynamics of emotional transformation [29].

Furthermore, qualitative investigation is essential to determine whether participants’ experiences correspond to the theoretical mechanisms proposed by EFT. The model posits that effective intervention produces increases in self-compassion and self-protection, decreases in self-criticism, and facilitates emotional transformation. Additionally, from the complementary perspective of CFT, intervention should produce decreased threat-system activation alongside increased soothing-system engagement. However, empirical verification of these theoretical predictions through examination of participants’ daily reflections has not been conducted.

A third gap concerns the granular emotional changes occurring during intervention. While EFT examines self-dialogue patterns and CFT addresses emotion regulation systems, neither framework has been applied to track shifts in discrete primary emotions across a compassion-focused intervention. No previous study has examined how Ekman's basic emotions change day-by-day during such training, missing an opportunity to provide convergent evidence for emotional transformation at the most fundamental level. The present study addresses these three gaps through triangulated analysis across EFT, CFT, and basic emotion theoretical frameworks.

We designed this investigation to provide a qualitative account of participants’ experiences during the EFT-HP intervention through a longitudinal examination of helping professionals’ daily experiences. By collecting and analyzing daily reflection data from 225 participants (with analyses focused on 146 completers), we aimed to examine whether participants’ lived experiences align with theoretical predictions and to characterize how emotional transformation unfolds across the 14-day intervention. Our analytical approach employed directed content analysis [30] with frequency quantification, integrating three complementary theoretical frameworks: the EFT model of emotional transformation [9], CFT's three emotion regulation systems [15,16], and Ekman's [18] basic emotion theory.

We pursued the following aims:

(a) to qualitatively characterize participants’ experiences of the EFT-HP intervention and indicators of its feasibility,
(b) to examine key EFT processes of self-criticism, self-compassion, and self-protection by developing detailed content descriptions of how participants experience emotional transformation in their daily reflections,
(c) to analyse key CFT processes of threat, soothing, and drive, and
(d) to analyze discrete primary emotions (fear, anger, sadness, disgust, contempt, surprise, joy, and love) to characterize the granular emotional shifts occurring across the intervention.

## Method

### Research design

This study employed a qualitative longitudinal design, collecting daily reflection data from helping professionals throughout The Emotion-Focused Training for Helping Professions (EFT-HP). The design enabled systematic analysis of how emotional transformation patterns shifted across the intervention trajectory, interpreted through three complementary theoretical frameworks: EFT's model of inner dialogue, CFT's three emotion regulation systems, and Ekman's basic emotion theory. While the primary analytical orientation was qualitative, frequency quantification of category distributions was incorporated to characterize temporal dynamics across the 14-day intervention period.

### Participants

Two hundred twenty-five helping professionals participated across three intervention cohorts in Slovakia. **Participants were recruited between 01/03/2023 and 30/03/2024.** Inclusion criteria comprised current employment in a helping profession (healthcare, social work, education, psychology, or related fields), at least one year of direct client/patient contact, Slovak language fluency, and internet access for daily engagement.

The sample was predominantly female (*n* = 198; 88.0%), reflecting the gender composition typical of helping professions in Central Europe. Ages ranged from 24 to 62 years (*M* = 38.7, *SD* = 9.4). Professional backgrounds included social work (32.4%), healthcare and nursing (24.0%), psychology and counseling (18.7%), education (15.1%), and other helping professions (9.8%). Participants averaged 11.3 years of professional experience (*SD* = 8.2, range: 1–38 years). Of the 225 participants who began the intervention and wrote the reflections, 146 (64.9%) completed at least 9 of the 14 days and were included in the primary analyses.

### Ethical considerations

The institutional review board of the Faculty of Social and Economic Sciences at Comenius University Bratislava approved the study (Protocol No. FSEV 1647/-4/2022/SD-CIII/1). All participants provided written online informed consent, including consent for anonymized data use in publications. All procedures were in accordance with the ethical standards of the institutional research committee and with the 1964 Helsinki Declaration and its later amendments.

**Use of Artificial Intelligence.** The qualitative coding in this study was AI-assisted. A large language model (Claude, Anthropic; December 2025) was used to assign each reflection unit to categories within the three frameworks (EFT inner dialogue, CFT emotion-regulation systems, and Ekman's discrete emotions) and to extract a verbatim supporting excerpt for every assigned code, retained in the coded dataset so that each code is auditable against the source. To evaluate the validity of the model’s output, the authors reviewed the AI-assigned codes against the pre-specified codebook definitions—one coder, with a second author serving as an independent auditor—verifying for every code that the retained verbatim excerpt supported the assigned category and resolving any discrepancies by consensus. A formal inter-rater reliability coefficient was not computed; the retention of a supporting excerpt for every code makes each classification independently auditable against the source text. The authors reviewed all AI-assisted output and take full responsibility for the analysis and its interpretation. AI tool usage affected only the qualitative coding of the daily reflection data: the assignment of reflection units to the EFT, CFT, and Ekman categories, the resulting category frequencies reported in the Results (Tables 1–3 and Figs 1–3), and the coded dataset derived from them. No other aspect of the study—its design, the intervention, data collection, the statistical and interpretive analyses, or the writing of this manuscript—was generated by an AI tool.

**Table 1:** Daily distribution of EFT inner-dialogue categories among cohort-1 completers (N = 7,307 coded reflection units); n (%), non-exclusive coding.

Day	Units	Self-Criticism *n (%)*	Self-Compassion *n (%)*	Self-Protection *n (%)*
1	553	79 (14.3)	106 (19.2)	22 (4.0)
2	515	46 (8.9)	105 (20.4)	32 (6.2)
3	524	30 (5.7)	100 (19.1)	27 (5.2)
4	534	72 (13.5)	124 (23.2)	37 (6.9)
5	535	24 (4.5)	103 (19.3)	32 (6.0)
6	533	34 (6.4)	71 (13.3)	60 (11.3)
7	532	10 (1.9)	90 (16.9)	24 (4.5)
8	520	15 (2.9)	106 (20.4)	16 (3.1)
9	513	16 (3.1)	75 (14.6)	18 (3.5)
10	493	10 (2.0)	35 (7.1)	12 (2.4)
11	519	15 (2.9)	54 (10.4)	24 (4.6)
12	513	5 (1.0)	81 (15.8)	19 (3.7)
13	507	10 (2.0)	156 (30.8)	6 (1.2)
14	516	20 (3.9)	100 (19.4)	26 (5.0)
**Total**	**7307**	**386 (5.3)**	**1306 (17.9)**	**355 (4.9)**

*Note.* Units represent discrete, semantically complete thoughts segmented from daily reflections. Categories are not mutually exclusive. Percentages represent the proportion of daily units containing each category. Completers only (*n* = 146 participants).

**Table 2:** Daily distribution of CFT emotion-regulation systems among cohort-1 completers (N = 7,307 coded reflection units); n (%), non-exclusive coding.

Day	Units	Threat *n (%)*	Drive *n (%)*	Soothing *n (%)*
1	553	196 (35.4)	109 (19.7)	208 (37.6)
2	515	115 (22.3)	111 (21.6)	222 (43.1)
3	524	115 (21.9)	101 (19.3)	180 (34.4)
4	534	110 (20.6)	115 (21.5)	167 (31.3)
5	535	91 (17.0)	84 (15.7)	207 (38.7)
6	533	154 (28.9)	89 (16.7)	141 (26.5)
7	532	53 (10.0)	106 (19.9)	140 (26.3)
8	520	57 (11.0)	95 (18.3)	212 (40.8)
9	513	43 (8.4)	137 (26.7)	134 (26.1)
10	493	19 (3.9)	120 (24.3)	101 (20.5)
11	519	44 (8.5)	98 (18.9)	126 (24.3)
12	513	23 (4.5)	102 (19.9)	128 (25.0)
13	507	25 (4.9)	111 (21.9)	196 (38.7)
14	516	36 (7.0)	115 (22.3)	162 (31.4)
**Total**	**7307**	**1081 (14.8)**	**1493 (20.4)**	**2324 (31.8)**

*Note.* Units represent discrete, semantically complete thoughts segmented from daily reflections. Categories are not mutually exclusive; units could activate multiple systems simultaneously. Percentages represent the proportion of daily units activating each system. Completers only (*n* = 146 participants).

**Table 3:** Daily Intervention Content and Corresponding Peaks in EFT and CFT Analyses.

Day	Exercise Content	EFT Peaks	CFT Peaks
1	Identifying signs of compassion fatigue	**Self-Compassion 19.2%**	**Soothing 37.6%**
2	Self-care modeled on client care	Self-Compassion 20.4%	Soothing 43.1% (overall peak)
3	Supervisor perspective letter	Self-Compassion 19.1%	Soothing 34.4%
4	Mindfulness sensory exercises	Self-Compassion 23.2%	Soothing 31.3%
5	Transforming self-critical voice	Self-Compassion 19.3%	Soothing 38.7%
6	Self-compassion toward vulnerable self	**Self-Compassion 13.3%**	Threat 28.9%
7	Standing up for oneself	Self-Compassion 16.9%	Soothing 26.3%
8	Letting go through self-soothing touch	**Self-Compassion 20.4%**	**Soothing 40.8%**
9	Professional mission and values	Self-Compassion 14.6%	Drive 26.7% (overall peak)
10	Non-mirroring for emotional regulation	**Self-Compassion 7.1%**	**Drive 24.3%**
11	Protection against compassion fatigue	Self-Compassion 10.4%	Soothing 24.3%
12	Work-life separation rituals	Self-Compassion 15.8%	Soothing 25.0%
13	Savoring the moment	**Self-Compassion 30.8% (overall peak)**	Soothing 38.7%
14	Work-life balance assessment and planning	Self-Compassion 19.4%	Soothing 31.4%

*Note.* Peaks indicate highest values; ↑ = notable increase; ↓ = notable decrease. Bold = highest or lowest value across entire intervention. SC = Self-Compassion; SP = Self-Protection; Crit = Self-Criticism.

**Fig 1:**
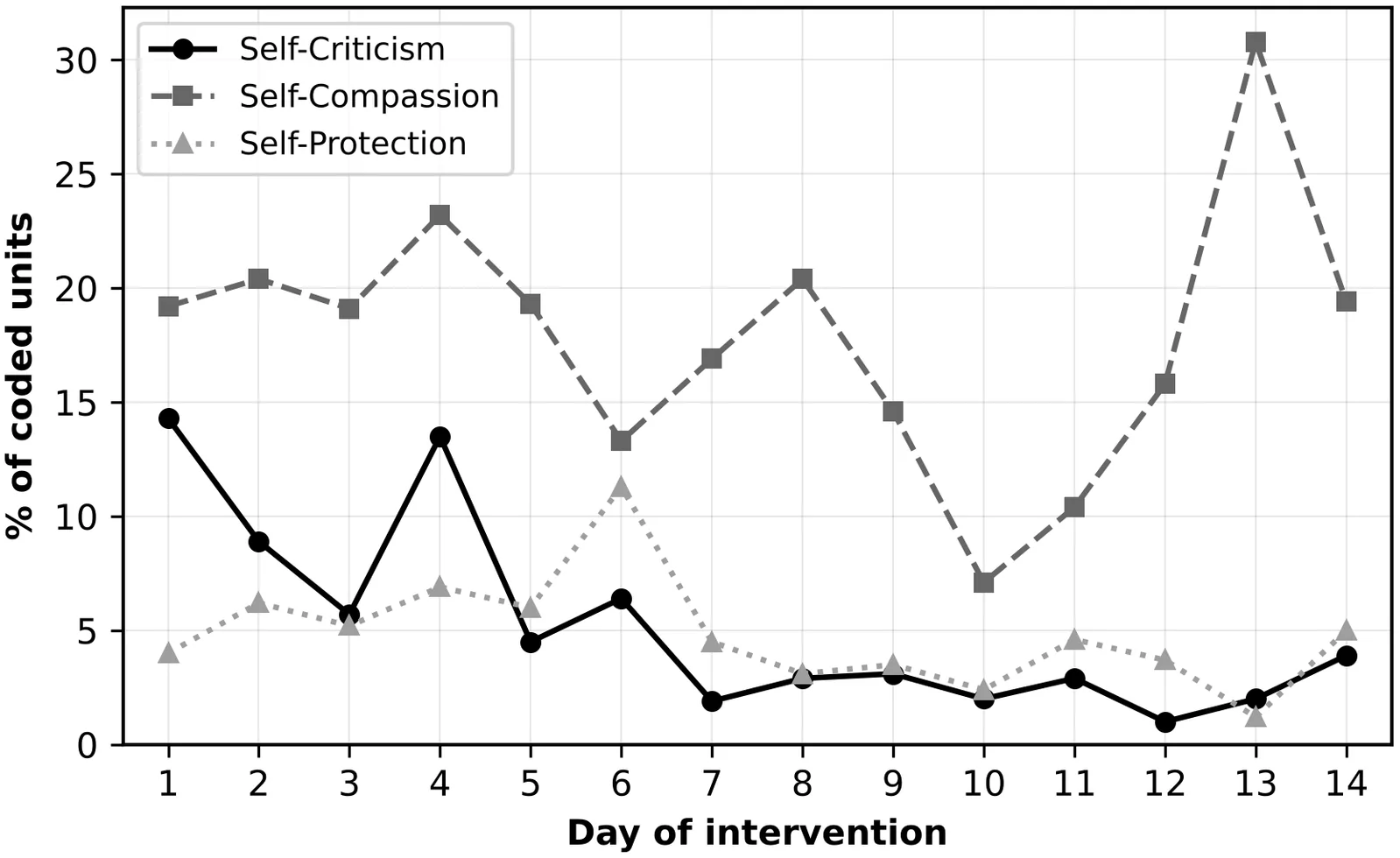
Daily Distribution of EFT Inner Dialogue Categories Across the 14-Day Intervention.

**Fig 2:**
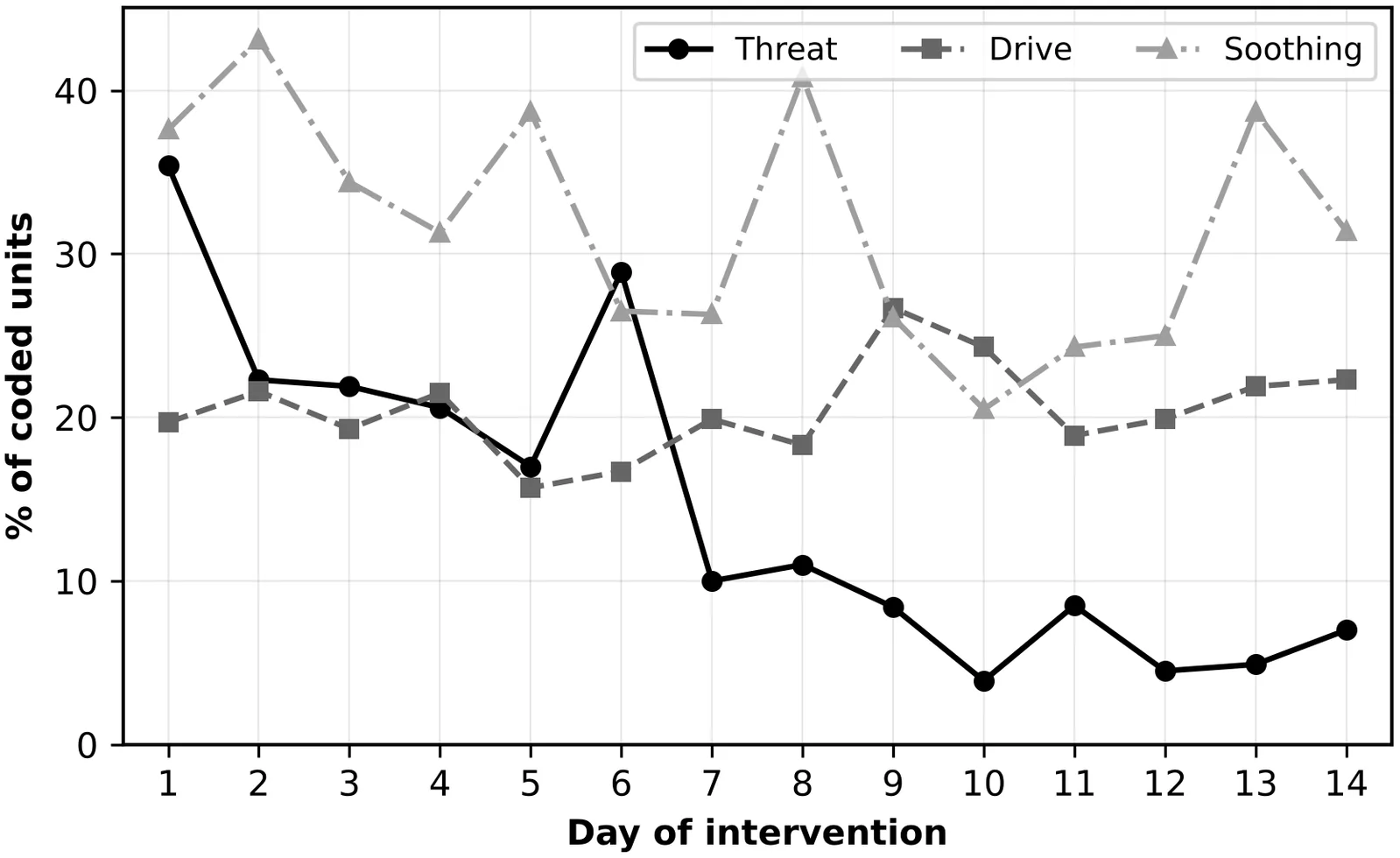
Temporal Dynamics of Emotion Regulation System Activation Across the 14-Day Intervention. Line chart depicting the proportional activation of CFT's three emotion regulation systems throughout the intervention period.

**Fig 3:**
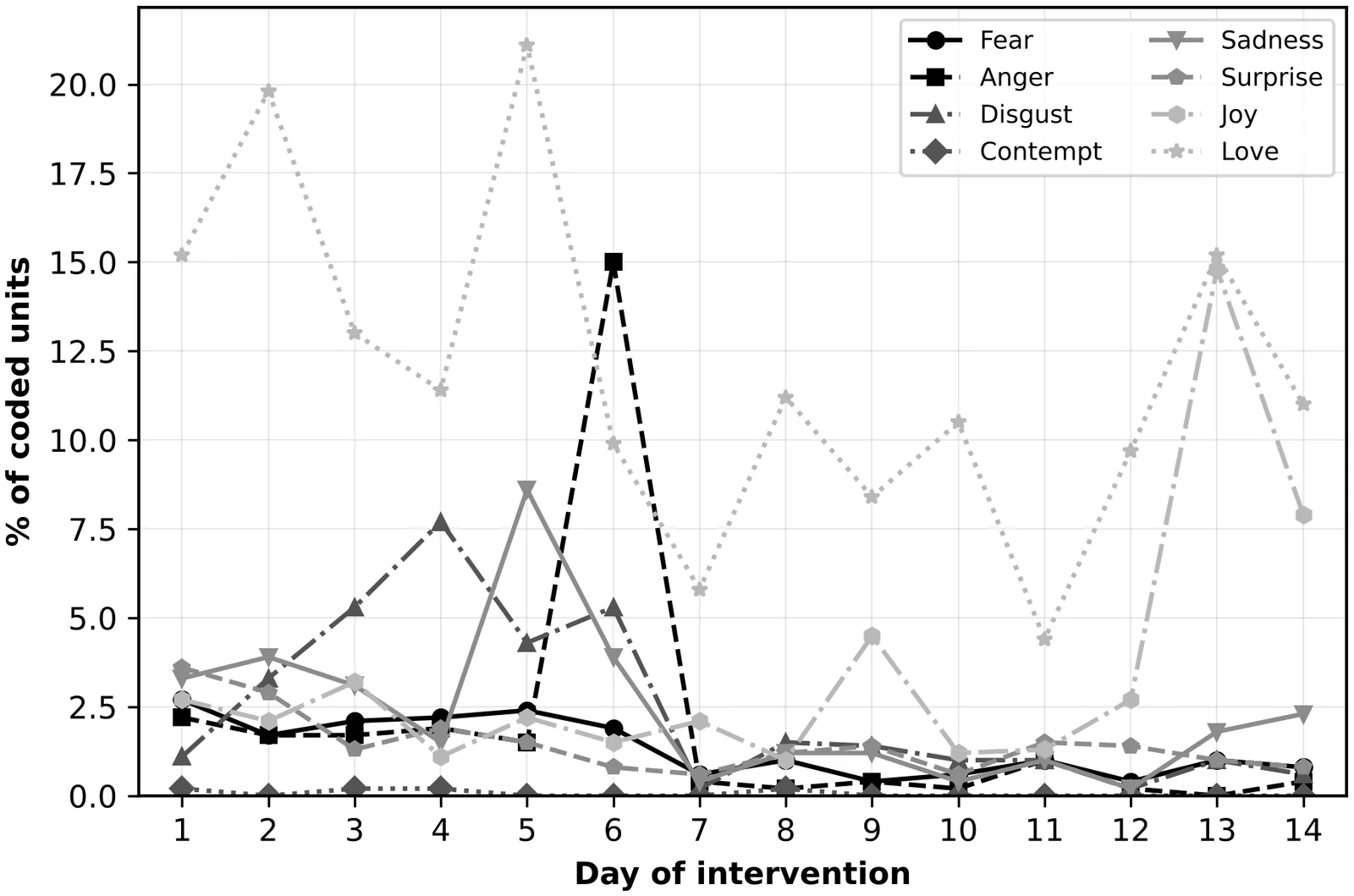
Primary Emotion Trajectories Across the 14-Day EFT-HP Intervention. Line chart depicting the proportional activation of eight primary emotions (Fear, Anger, Disgust, Contempt, Sadness, Surprise, Joy, and Love) throughout the intervention period.

### Intervention

The Emotion-Focused Training for Helping Professions (EFT-HP) is a 14-day online asynchronous intervention designed to address compassion fatigue, enhance compassion satisfaction, and reduce self-criticism among helping professionals [26,27]. The intervention is grounded in the evidence-based model of change from Emotion-Focused Therapy [9,12,13] and incorporates techniques from Somatic Trauma Therapy [31]. The training develops both self-compassion and self-protection skills, recognizing that effective intervention for self-criticism must include not only soothing self-compassion but also assertive/self-protective responses that enable individuals to stand up for themselves [9,12].

Participants receive daily exercises delivered via email, with each exercise requiring approximately 15 minutes to complete. The intervention content encompasses exercises targeting compassion fatigue awareness, cultivating compassion for others and self-compassion, combating self-criticism, promoting self-care, clarifying professional mission, improving work-life balance, and strengthening trauma protection skills essential for helping professionals [26].

Across the 14 days, the programme follows a structured arc: it opens by building awareness of compassion fatigue, moves through exercises cultivating compassion for others and self-compassion, and introduces work on self-criticism and self-protection (learning to stand up for oneself) around the middle of the programme (for example, boundary-setting and self-protection exercises around Days 6–7 and a soothing-touch and self-care exercise around Day 8), before consolidating gains through self-care, work–life balance, savouring and gratitude, and trauma-protection exercises in the closing days (for example, gratitude and savouring around Day 13). A day-by-day summary of the module content is provided in Table 3, so that the treatment is fully specified before the analysis of daily reflections is presented.

### Procedure and data collection

Following ethical approval and informed consent, participants completed baseline measures of compassion satisfaction, compassion fatigue, self-criticism, and the three flows of compassion. They then received daily instructions via email. Each morning, a new module became available, with email reminders encouraging completion within 24 hours. Post-intervention assessments followed Day 14, with follow-up measurement two months after the intervention.

Participants submitted daily written reflections following each module. Prompts varied to align with module content while maintaining focus on personal experience. Participants responded in Slovak with no length requirements. Reflections ranged from brief (2–3 sentences) to extended narratives. The unit of analysis was the individual reflection response. For the 146 completers included in the primary analyses, this yielded 7,307 coded reflection units.

### Researcher positionality

Consistent with qualitative content analysis methodology, we acknowledge that our theoretical commitments shaped the analytical process. Team members had training in Emotion-Focused Therapy and Compassion-Focused Therapy, influencing both intervention design and interpretation. All coauthors also had professional experience in helping fields, providing insider perspective while potentially shaping what we noticed.

### Data analysis

We analyzed participant reflections using directed content analysis [30] with frequency quantification within an integrative theoretical framework combining Emotion-Focused Therapy [9], Compassion-Focused Therapy [15,16], and Ekman's [18] basic emotion theory. This hybrid approach enabled systematic classification of experiential content according to theoretically grounded emotional processes while remaining sensitive to emergent patterns in participants’ daily narratives. The analytic framework was particularly suited to capturing processes of emotional transformation, central to EFT, as they unfolded across the intervention.

We selected directed content analysis with frequency quantification for several reasons [30]: its systematic procedures support transparency in large datasets; its theoretical flexibility permits integration of deductive frameworks; and its quantification of category distributions enables temporal comparison across intervention days. Consistent with directed content analysis methodology [30], we employed a deductive approach using theoretically pre-defined categories rather than generating themes inductively.

Throughout the analysis, reported percentages represent the proportion of coded reflection units expressing a given category on a given day, aggregated across participants, rather than participant-level scores. Accordingly, statements describing increases or decreases (for example, the 73% relative reduction in Self-Criticism) refer to changes in the prevalence of coded content across the corpus and should not be interpreted as psychometric change within individuals.

### Unit of analysis and classification structure

The unit of analysis was the individual reflection response—a participant's answer to one reflection prompt on one day. Each unit was coded within the three frameworks described below; codes were assigned non-exclusively, so a unit could receive more than one category when its content warranted (for example, self-criticism alongside emerging self-compassion). For the 146 completers, the corpus comprised 7,307 coded reflection units.

Three complementary analytical frameworks were applied. For the EFT analysis, units were coded into three categories based on Emotion-Focused Therapy's model of inner dialogue: Self-Criticism (negative inner dialogue), Self-Compassion (positive soothing dialogue), and Self-Protection (assertive dialogue). For the CFT analysis, units were coded according to the three emotion regulation systems: Threat, Drive, and Soothing. For Ekman’s basic emotions [18] analysis unites were coded into fear, anger, disgust, contempt, sadness, surprise, and joy expressions. Categories were coded non-exclusively, allowing units to be assigned to multiple categories when appropriate. This approach captures the complexity of emotional experience, as participants’ reflections often simultaneously expressed multiple processes (e.g., self-criticism alongside emerging self-compassion) consistent with EFT's sequential model of emotional transformation [11] and CFT's conceptualization of interacting emotion regulation systems [16]. Besides, basic emotion theory recognizes that multiple discrete emotions frequently co-occur within emotional episodes and that emotional blends are common in naturalistic experience [18,32]. This approach captures the complexity of emotional experience while enabling temporal comparison.

Coding was AI-assisted and evidence-based: each reflection unit was assigned to categories within the three frameworks, and a verbatim supporting excerpt was recorded for every assigned code (see Use of Artificial Intelligence). Category definitions and decision rules for each framework were specified before coding and documented in a codebook. One human coder, with a second author serving as an independent auditor, reviewed the coded output. A formal inter-rater reliability coefficient was not computed; instead, each assigned code was recorded together with a verbatim supporting excerpt so that every classification can be audited against the source text. Disagreements identified during review were resolved through discussion and consensus. Reflexivity was maintained through team discussion of how clinical training and professional experience might shape categorization (see Researcher positionality).

## Results

### Emotion-focused theory analysis

Reflections from the 146 participants who completed at least 9 of the 14 days of the EFT-HP intervention formed the corpus for the primary analyses (7,307 coded reflection units). Each unit was coded, with a verbatim supporting quotation retained for every assigned category, into three theoretically derived inner-dialogue categories from Emotion-Focused Therapy: Self-Criticism (negative, self-punitive, self-ruminating inner dialogue), Self-Compassion (self-soothing, self-kind, self-caring inner dialogue), and Self-Protection (assertive dialogue, boundary-setting, and standing up for oneself). Categories were not mutually exclusive.

Across the intervention, Self-Compassion was the most frequently expressed inner-dialogue category, appearing in 17.9% of coded units (n = 1306), followed by Self-Criticism at 5.3% (n = 386) and Self-Protection at 4.9% (n = 355). The daily distribution revealed distinct temporal patterns consistent with EFT theoretical predictions (see Table 1 and Fig 1).

Self-Criticism decreased across the intervention, from 14.3% of coded units on Day 1 to 3.9% on Day 14, a 73% relative decrease. The trajectory indicates an early and sustained move away from harsh self-evaluation as participants engaged with the intervention.

Self-Compassion was the dominant and most stable inner-dialogue category, present at 19.2% on Day 1 and 19.4% on Day 14, and peaking on Day 13 (30.8%) during the savoring and appreciation module. Participants maintained and, on specific days, markedly heightened their capacity for self-soothing dialogue.

Self-Protection was comparatively infrequent overall (4.9%) but showed a pronounced, content-specific peak on Day 6 (11.3%)—the module devoted to protective anger and standing up for oneself, from which the study takes its name. From Day 1 (4.0%) to Day 14 (5.0%) it remained broadly stable, with the Day-6 spike demonstrating that the targeted exercise activated assertive, boundary-setting dialogue.

These patterns are consistent with Emotion-Focused Therapy's model of adaptive emotional processing: a shift away from self-criticism, sustained self-compassion, and targeted activation of self-protection when the intervention addressed it directly. Because the analysis is observational and single-group, the patterns describe participants’ reflections rather than establishing that the intervention caused the change.

Line chart of the daily prevalence of three inner-dialogue categories. Self-Criticism declines from 14.3% (Day 1) to 3.9% (Day 14); Self-Compassion is sustained and peaks on Day 13 (30.8%); Self-Protection peaks on Day 6 (11.3%). Non-exclusive coding. n = 146 completers; N = 7,307 coded units.

### Illustrative participant statements for EFT inner dialogue categories

*Self-Criticism:*

*“I keep telling myself that I should be stronger, that I shouldn’t feel this way. Other colleagues handle it better—why can’t I?“ (Day 1)*

*“I noticed how harsh my inner voice is. It tells me I’m not good enough, that I’m failing my clients when I feel tired.“ (Day 3)*

*Self-Compassion:*

*“Today I realized that I deserve the same kindness I give to my clients. It was like giving myself permission to be human.“ (Day 5)*

*“I felt warmth toward myself for the first time in a long time. Like wrapping myself in a soft blanket of understanding.“ (Day 8)*

*Self-Protection:*

*“I imagined standing up for that vulnerable part of me and saying No, you cannot speak to her that way. It felt empowering.“ (Day 7)*

*“I set a boundary with a colleague today. I said no without guilt. The exercise helped me see that protecting myself is not selfish.“ (Day 10)*

These accounts illustrate the developing capacity for self-compassionate awareness and the integration of self-compassion and self-protection into daily life to deal with self-criticism, precisely what the intervention aimed to cultivate.

### Compassion-Focused Therapy (CFT) Analysis

Analysis of the 7,307 coded reflection units from completers (n = 146) revealed clear patterns of emotion-regulation-system activation consistent with CFT's three-systems framework. Across the whole corpus, the soothing system was the most frequently coded of the three systems (n = 2324; 31.8% of units), followed by the drive system (n = 1493; 20.4%), with the threat system least represented (n = 1081; 14.8%). Codes were assigned non-exclusively (see Table 2 and Fig 2).

### Temporal dynamics

Day-by-day analysis revealed a marked shift in system activation. The threat system decreased substantially, from 35.4% on Day 1 to 7.0% on Day 14, a 80% relative decrease, with most of the reduction occurring over the first days of the intervention.

Over the course of the intervention the soothing system fluctuated from day to day—37.6% on Day 1, a Day-2 peak of 43.1%, and 31.4% on Day 14—without a systematic upward or downward trend; it nonetheless remained the most prevalent of the three systems on almost every day.

The drive system was stable to slightly increasing, from 19.7% on Day 1 to 22.3% on Day 14 (peak on Day 9, 26.7%), indicating that self-compassion cultivation did not suppress goal-directed engagement.

The clearest pattern in the data is the threat-to-soothing shift: as threat-system content fell across the fortnight, soothing content remained the most prevalent system, consistent with the intervention's orientation toward affiliative, self-soothing processing.

### Illustrative participant statements for Gilbert’s three systems

*Threat System:*

*“I feel anxious when I think about returning to work tomorrow. The weight of my clients’ problems sits on my chest like a stone.“ (Day 1)*

*“There is fear in me that I am not doing enough, that I am somehow failing everyone who depends on me.“ (Day 4)*

*Drive System:*

*“I feel motivated to implement what I learned today. I have a clear plan for how to take better care of myself.“ (Day 9)*

*“I want to achieve a better balance. I’m setting concrete goals for my self-care routine.“ (Day 14)*

*Soothing System:*

*“A deep sense of calm washed over me during the exercise. I felt safe and content, like everything would be okay.“ (Day 2)*

*“When I touched my heart and spoke kindly to myself, I felt a warmth spread through my body. Peace. Finally, peace.“ (Day 8)*

In Fig 2, the threat system declines from 35.4% (Day 1) to 7.0% (Day 14), a 80% relative decrease; the soothing system remains the most prevalent system on almost every day (peak Day 2, 43.1%); the drive system is stable (15.7%–26.7%). Non-exclusive coding. n = 146 completers; N = 7,307 coded units.

The discrete-emotion analysis complements the EFT and CFT findings. Because most reflection units did not name a specific discrete emotion, absolute prevalences are lower than for the broader inner-dialogue and emotion-system categories; the direction of change, however, mirrors the other two frameworks.

In Fig 3, Fear declines from 2.7% (Day 1) to 0.8% (Day 14); Anger peaks on Day 6 (15.0%) during the protective-anger module and falls to 0.4% by Day 14; Joy rises to a Day-13 peak (14.8%); and Love is comparatively prominent, peaking on Day 5 (21.1%). Non-exclusive, AI-assisted coding with retained evidence. n = 146 completers; N = 7,307 coded units.

Among positive emotions, Joy increased from 2.7% on Day 1 to 7.9% on Day 14, peaking on Day 13 (14.8%) during the savoring module. Love was the most frequently expressed discrete emotion overall (11.9%; n = 872) and peaked on Day 5 (21.1%) during the self-compassion module.

Negative discrete emotions were low and declined. Fear decreased from 2.7% to 0.8% (71% relative decrease). Anger peaked on Day 6 (15.0%)—the protective-anger and boundary-setting module—before declining to 0.4% by Day 14, suggesting the exercise provided a constructive outlet for anger. Sadness peaked on Day 5 (8.6%); Disgust, Surprise, and Contempt remained low throughout.

To complement the EFT and CFT analyses, each unit was coded for Ekman's discrete emotions (Anger, Fear, Sadness, Joy, Disgust, Surprise, Contempt) together with Love, using the same non-exclusive, evidence-based procedure. As with the other frameworks, the coding was AI-assisted and every assigned emotion carries a verbatim supporting excerpt.

### Day-by-day analysis: Linking intervention content to observed patterns

The temporal peaks in all three frameworks align with the corresponding module content: Self-Protection and Anger peak on Day 6 (protective anger); Love peaks on Day 5 (self-compassion); and Self-Compassion and Joy peak on Day 13 (savoring). Table 3 summarizes these correspondences.

### The temporal picture

At the daily level, the peaks map onto the intervention's structure. Threat and self-criticism are highest at the outset and decline as the programme proceeds; soothing and self-compassion remain the most prevalent categories on most days; and framework-specific peaks coincide with the modules that target them—Self-Protection and Anger on Day 6 (protective anger), Love on Day 5 (self-compassion), and Self-Compassion (30.8%) and Joy on Day 13 (savoring). By Day 14, threat (7.0%) and self-criticism (3.9%) are low while soothing, drive, and self-compassion remain high, indicating an integrated profile at the end of the intervention.

### Integration: The temporal architecture of emotional transformation

These findings reveal an intentional temporal architecture following a theoretically coherent sequence: (1) awareness and threat activation through compassion fatigue identification, (2) rapid soothing engagement through redirecting existing compassion skills, (3) cognitive work to transform self-critical dialogue, (4) accessing vulnerability with protective assertiveness, (5) embodied self-soothing, (6) professional boundary skills, and (7) consolidation through positive emotion amplification and future planning. The discrete emotion analysis provides convergent evidence: Fear declined 91% across the intervention, Anger peaked strategically during self-protection exercises (Day 6), Love peaked during compassion-focused days (Days 5 and 8), and Joy peaked during the savoring exercise (Day 13). This sequence mirrors Pascual-Leone and Greenberg's [11] sequential model of emotional processing: movement from global distress through painful emotions toward adaptive emotions.

## Discussion

This study pursued four aims: (a) to qualitatively characterize participants’ experiences of the EFT-HP intervention and indicators of its feasibility, (b) to examine key EFT processes of self-criticism, self-compassion, and self-protection, (c) to analyse key CFT processes of threat, soothing, and drive, and (d) to analyze discrete primary emotions to characterize the granular emotional shifts occurring across the intervention.

Regarding aim (a), the findings speak to the feasibility and acceptability of the EFT-HP intervention. Of 225 participants who began the intervention, 146 (64.9%) completed at least 9 of the 14 days, generating 7,307 coded reflection units. The substantial completion rate and rich qualitative data support the intervention's acceptability among helping professionals.

Regarding aims (b) and (c), analyses revealed substantial shifts consistent with EFT and CFT theoretical predictions. Self-Criticism declined from 14.3% to 3.9% (73% relative decrease), while Self-Compassion was sustained throughout and peaked on Day 13 (30.8%). The CFT threat system decreased from 35.4% to 7.0% (80% relative decrease), while the soothing system remained the most prevalent system on almost every day (peak on Day 2, 43.1%). These convergent patterns across two complementary frameworks, applied to the same reflections, provide a coherent picture of emotional transformation.

Regarding aim (d), the discrete-emotion analysis showed Fear declining from 2.7% to 0.8% (71% relative decrease) and Anger peaking on Day 6 (15.0%) before falling to 0.4%, while Joy rose to a Day-13 peak (14.8%). These discrete-emotion shifts, though modest in absolute terms, parallel the threat-to-soothing pattern seen in the EFT and CFT analyses.

From the EFT perspective, the findings are consistent with the emotional transformation theorized by Greenberg [9] and Pascual-Leone and Greenberg [11]. Self-Criticism showed a substantial decline (73% decrease from Day 1 to Day 14), reflecting a shift of harsh self-evaluative dialogue toward more compassionate self-relating. Self-Compassion was the dominant inner-dialogue category throughout and peaked on Day 13 (30.8%), indicating activation of adaptive self-compassionate dialogue. Self-Protection was infrequent overall but peaked on Day 6 (11.3%), the protective-anger module, suggesting that participants engaged the capacity to assertively stand up for themselves against the inner critic when the intervention addressed it directly [12,13].

From the CFT perspective, the overall predominance of soothing-system content (31.8% of coded units) alongside the 80% relative decline in threat-system content is consistent with the intervention orienting participants toward affiliative, self-soothing emotion regulation.

From Ekman's basic emotion perspective, the discrete emotion analysis is consistent with the transformation captured by the EFT and CFT frameworks, although discrete emotions were expressed less frequently. Fear declined from 2.7% to 0.8% (71% relative decrease), paralleling the threat-system reduction observed in the CFT analysis. Anger peaked on Day 6 (15.0%) during the protective-anger exercise, aligning with EFT's emphasis on assertive self-protection. Love was the most prominent discrete emotion (11.9% overall) and peaked on Day 5 (21.1%) during compassion-focused exercises, and Joy peaked on Day 13 (14.8%) during savoring. This coherence across the three frameworks—Fear paralleling threat, Love paralleling soothing, and Anger aligned with self-protection—reflects analytic (theoretical) triangulation applied to the same reflection data; because the frameworks were not independent data sources, their agreement increases interpretive coherence rather than providing independent confirmation.

### Theoretical contributions

These findings contribute to theoretical understanding in several ways. Most fundamentally, they provide empirical support for the Emotion-Focused Therapy model of emotional transformation, with Compassion focused therapy model [16] offering complementary support for understanding EFT-HP intervention processes. The demonstrated utility of these frameworks for organizing and interpreting daily experiences suggests their value for future qualitative research in this domain.

The finding that drive remained stable while its qualitative character shifted suggests that the relationship among systems is not purely competitive (cf. [33]). Rather than soothing suppressing drive, a well-developed soothing system appears to provide a foundation from which healthy drive can operate—what might be termed synergistic regulation. This finding aligns with EFT's emphasis on emotional transformation through accessing adaptive emotions, with CFT's [16] model providing a complementary framework for understanding how system balance involves not just relative activation levels but relational configurations.

The discrete emotion analysis, grounded in Ekman's [18,19] basic emotion theory, is consistent with both the EFT and CFT frameworks. The 71% reduction in fear-related content parallels the threat-system decline observed in the CFT analysis, so that these theoretically distinct constructs—fear as a primary emotion and threat as a regulatory system—show corresponding patterns. Because the frameworks were applied to the same reflections rather than to independent data, this convergence is best understood as analytic (theoretical) triangulation that strengthens interpretive coherence rather than providing independent confirmation. The prominence of love (peaking on Day 5, 21.1%) alongside the overall predominance of the soothing system is consistent with the theoretical link between affiliative positive emotions and the soothing system [16].

The present findings provide strong support for core principles of Emotion-Focused Therapy. The sequential model of emotional processing proposed by Pascual-Leone and Greenberg [11] predicts that adaptive emotions such as self-compassion and self-protection emerge through accessing and processing core painful emotions. Our day-by-day analysis revealed precisely this pattern: participants moved from initial threat-dominated processing (Day 1) through engagement with difficult self-related content (Day 6) toward increasingly stable soothing-system activation, with the self-protection exercise on Day 7 producing striking regulatory effects. This provides qualitative evidence for the EFT model of emotional transformation in a daily intervention context.

### Effectiveness and feasibility of the EFT-HP intervention

The present qualitative findings complement and extend the quantitative evidence for EFT-HP effectiveness reported in two previous randomized controlled trials. Halamová et al. [26] demonstrated that participants completing the 14-day EFT-HP intervention showed significant reductions in secondary traumatic stress, burnout, and self-criticism, along with significant increases in self-compassion, compared to a control group. These effects were maintained at 2-month follow-up, suggesting durable benefits. More recently, Halamová et al. [27] replicated these findings in a larger sample, demonstrating significant improvements in compassion satisfaction alongside reductions in compassion fatigue.

Our qualitative analysis enriches these quantitative outcomes by describing the daily patterns that accompany such changes. From the EFT perspective, the 73% reduction in Self-Criticism from Day 1 to Day 14 is consistent with the quantitative reductions in self-criticism reported by Halamová et al. [26,27]. The sustained predominance of Self-Compassion, peaking on Day 13 (30.8%), aligns with the increases in self-compassion documented in both RCTs. The content-specific peak of Self-Protection on the boundary-setting day is consistent with the assertive self-care capacities emphasized in EFT's model [11].

From the CFT perspective, the 80% reduction in threat-system content from Day 1 to Day 14 is consistent with the quantitative reductions in secondary traumatic stress and burnout reported by Halamová et al. [26,27]. Similarly, the overall predominance of soothing-system content (31.8% of coded units) aligns with the increases in self-compassion documented in both RCTs.

From Ekman's basic emotion perspective, the 71% reduction in Fear (from 2.7% to 0.8%) is consistent with threat reduction, while the peaks in Love (Day 5) and Joy (Day 13) indicate activation of positive emotional states associated with compassion satisfaction. These discrete emotion trajectories are consistent with the emotional-transformation processes proposed by EFT-HP; however, as an uncontrolled, single-group qualitative study, they cannot establish that the intervention caused these changes or distinguish emotional transformation from symptom suppression.

Importantly, the present study demonstrates that participants actively engaged with the intervention content in meaningful ways, producing over 22,000 analyzable reflection units—evidence of both feasibility and participant engagement that standardized measures cannot capture.

The feasibility of the EFT-HP intervention is further supported by the daily engagement patterns observed. Of the 225 participants who began the intervention, 146 (64.9%) completed at least 9 of the 14 days, and these completers consistently submitted reflections throughout the period. The brief, asynchronous format appeared to accommodate the demanding schedules of many helping professionals. At the same time, because 35.1% of those who began did not complete at least 9 of the 14 days, completion was not universal, and feasibility indicators based on completers may overstate the intervention’s acceptability. This finding addresses a critical gap identified in the intervention literature: many evidence-based programs require 8 weeks of in-person attendance, which helping professionals often cannot accommodate due to shift work, caregiving responsibilities, and the emotional demands of their roles [34].

### Practical implications

For clinicians designing interventions similar to EFT-HP, these findings offer several actionable insights. The rapid early response (Day 1 to Day 2) suggests that initial content should directly address threat-based processing to capitalize on participants’ readiness for change (cf. [35]). The pronounced Day 7 effects highlight the importance of self-protection exercises (learning to stand up for oneself), optimally positioned mid-intervention when foundational concepts are established [36]. The Day 6 pattern, temporary threat increases when practicing self-compassion toward vulnerable oneself, suggests that such content requires careful framing and follow-up. Participants should be prepared for temporary discomfort, with subsequent sessions providing restoration. The overall 14-day format appears viable for producing meaningful change, offering an alternative to longer programs that many helping professionals cannot accommodate [34].

For organizations employing helping professionals, the demonstrated effectiveness of a brief online intervention suggests feasible integration into professional development, continuing education, or employee wellness initiatives. The modest time requirement (15–25 minutes daily) and technology-delivered format address accessibility barriers that limit participation in traditional programs [37]. The diversity of participants across professional fields and the consistency across three cohorts are encouraging; however, the predominantly female and entirely Slovak sample, together with the single-group design, means that transferability to other helping professions and cultural contexts remains to be established.

### Limitations

Several limitations warrant consideration. The single-group design without control condition means we cannot definitively attribute changes to the intervention versus other factors—regression to the mean, passage of time, or external events. The temporal patterns aligned with intervention content provide suggestive evidence, but randomized controlled designs would strengthen causal inference.

Reliance on self-report reflection data introduces potential biases. Participants may have emphasized content consistent with perceived expectations or most salient at the time of writing. Additionally, the analyses are restricted to completers (146 of 225; 64.9%), which could introduce selection bias. To assess this, we compared completers with the 79 non-completers on baseline characteristics. The groups did not differ significantly on age, gender, education, years of professional experience, or use of supervision, or on any baseline measure of self-criticism, secondary traumatic stress, burnout, or the three flows of compassion (all p > .17); completers reported somewhat more weekly client-contact hours (M = 23.5 vs. 18.6; p = .013, d = 0.36), a difference that did not survive correction for multiple comparisons (see S1 Table). Attrition therefore appeared largely unrelated to baseline characteristics, although unmeasured differences cannot be excluded, and the findings describe the experiences of those who engaged substantially with the programme.

The predominantly female (88.0%), entirely Slovak sample limits generalizability across genders and cultures. While the gender composition reflects Central European helping professions, findings may not extend to male-dominated fields or cultures with different orientations toward self-compassion.

We did not include follow-up assessment in the present study to determine whether observed changes persisted. The sustainability of emotion regulation shifts remains an important question. Finally, although the coding framework was theoretically pre-defined and applied through the consensus procedures described in the Method, we did not compute a formal inter-rater reliability coefficient; the reported frequencies therefore reflect the research team’s shared analytic lens and should be interpreted as interpretive classifications rather than psychometric measurements.

### Future directions

These findings suggest several research priorities. Randomized controlled trials comparing this intervention to active controls would establish efficacy and identify specific active ingredients. Longitudinal studies with follow-up at 1, 3, and 6 months would address sustainability questions. Component analysis could identify which elements most influence each system—useful for intervention optimization.

Research examining individual differences in response would enhance understanding of for whom self-compassion training is most beneficial. Variables such as baseline self-compassion, attachment style, and severity of occupational stress may moderate effects. Cross-cultural replication would clarify cultural boundaries on the patterns observed here. Mixed-methods designs combining daily reflection analysis with standardized measures and physiological indicators would provide converging evidence. Implementation research examining integration into organizational contexts would facilitate translation into practice.

## Conclusion

This study provides the first large-scale qualitative examination of helping professionals’ daily experiences during the Emotion-Focused Training for Helping Professions (EFT-HP), analyzed through both Emotion-Focused Therapy's model of inner dialogue and CFT's [15,16] three regulation systems framework. The findings show that, across the intervention, participants’ daily reflections shifted substantially in emotional processing, marked by significant self-criticism and threat reduction, sustained self-compassion and soothing activation, and maintained healthy drive and self-protection. The discrete emotion analysis further strengthened these findings, revealing a large reduction in negative emotions and a large increase in positive emotions over the course of the intervention, providing convergent, analytically triangulated description across three frameworks that is consistent with the processes proposed by EFT-HP, although this observational evidence cannot on its own establish intervention effectiveness. These patterns align with both EFT and CFT theoretical mechanisms while revealing the temporal dynamics through which emotion regulation shifts unfold during the intervention.

For helping professionals confronting the occupational hazards of compassion fatigue, burnout, and secondary traumatic stress, self-compassion offers a pathway toward sustainable well-being. What these data reveal is that cultivating self-compassion involves not merely feeling better but developing fundamentally different ways of relating to oneself during difficulty—shifting from threat-based self-criticism to soothing-based self-kindness while maintaining the motivation necessary for continued engagement. As demands on helping professionals intensify, the need for effective, accessible interventions supporting their well-being becomes ever more pressing. This study contributes to that effort by illuminating how the EFT-HP intervention can be helpful for both personal flourishing and professional sustainability.

## Supporting information

S1 Table Baseline comparison of the 146 completers and the 79 non-completers on demographic and psychological characteristics, with tests of difference. (DOCX)
