# Supplementary material for: Standing up heals: A qualitative analysis of participants' daily reflections from Emotion-Focused Training for Helping Professions (EFT-HP)
Source: PLoS One. 2026 Oct 29;21(10):e0354851. doi: 10.1371/journal.pone.0354851 (PMC13718924; doi:10.1371/journal.pone.0354851)
Supplement: Supplementary file 1 [file pone.0354851.s001.docx]

**S1 Table.** Baseline comparison of completers and non-completers.

| **Baseline characteristic** | **Completers (n = 146)** | **Non-completers (n = 79)** | ***p*** |
| --- | --- | --- | --- |
| Age, years | 39.0 (10.8) | 38.6 (11.2) | .82 |
| Years of professional experience | 12.5 (10.5) | 11.1 (8.8) | .28 |
| Weekly client-contact hours | 23.5 (13.9) | 18.6 (13.1) | .013 |
| Women, % | 85.9 | 86.1 | 1.00 |
| Master’s degree or higher, % | 84.5 | 84.8 | .77 |
| Currently in supervision, % | 32.9 | 43.0 | .17 |
| ***Baseline measures, M (SD)*** |  |  |  |
| Self-criticism (FSCRS total) | 32.3 (11.5) | 33.9 (9.5) | .28 |
| Secondary traumatic stress | 19.4 (7.5) | 19.5 (6.9) | .89 |
| Burnout | 20.4 (7.0) | 21.1 (5.5) | .45 |
| Self-compassion (SOCS-S) | 74.9 (16.2) | 75.2 (10.2) | .88 |
| Compassion for others (SOCS-O) | 79.8 (15.3) | 79.9 (7.9) | .96 |
| Compassion from others (SOCS-FO) | 92.7 (19.9) | 90.5 (16.3) | .39 |

*Note.* Completers (n = 146) were participants who completed at least 9 of the 14 days of daily reflections; non-completers (n = 79) completed fewer than 9 days. Continuous variables are reported as M (SD) and compared with Welch’s t-test (Mann–Whitney U tests were concordant on every variable); categorical variables were compared with the chi-square test. Ns for continuous demographic variables vary slightly because of item-level missing data (age n = 218; years of experience n = 215; client-contact hours n = 207). FSCRS = Forms of Self-Criticizing/Attacking and Self-Reassuring Scale; SOCS = Sussex-Oxford Compassion Scale (S = for the self, O = for others, FO = from others). Completers and non-completers did not differ significantly on any baseline demographic or psychological variable except weekly client-contact hours (Cohen’s d = 0.36), and that difference did not survive Holm correction for the 12 comparisons (adjusted p ≈ .16).
